# Sulforaphane as a potential therapeutic agent: a comprehensive analysis of clinical trials and mechanistic insights

**DOI:** 10.1017/jns.2025.10033

**Published:** 2025-09-16

**Authors:** Atsushi Saito, Shoichi Ishikawa, Kun Yang, Akira Sawa, Koko Ishizuka

**Affiliations:** 1 Department of Neuroscience, Johns Hopkins University School of Medicine, Baltimore, MD, USA; 2 Department of Psychiatry, Johns Hopkins University School of Medicine, Baltimore, MD, USA; 3 Department of Pharmacology and Molecular Sciences, Johns Hopkins University School of Medicine, Baltimore, MD, USA; 4 Department of Genetic Medicine, Johns Hopkins University School of Medicine, Baltimore, MD, USA; 5 Department of Biomedical Engineering, Johns Hopkins University Whiting School of Engineering, Baltimore, MD, USA; 6 Department of Mental Health, Johns Hopkins Bloomberg School of Public Health, Baltimore, MD, USA

**Keywords:** Clinical trials, Intervention, Mechanisms, Sulforaphane, SFN, Sulforaphane, Keap1, Kelch-like ECH-associated protein-1, Nrf2, Nuclear factor erythroid 2-related factor 2, HDAC, Histone deacetylase, NQO1, NAD(P)H quinone dehydrogenase 1, HO-1, Heme oxygenase 1, BMI, Body mass index, GSTM1, Glutathione S-transferase Mu 1, ASD, Autism spectrum disorder, FXTAS, Fragile-X-associated tremor and ataxia syndrome, SZ, Schizophrenia

## Abstract

Sulforaphane (SFN), a bioactive compound derived from glucoraphanin in cruciferous vegetables such as broccoli, has been extensively studied for its therapeutic potential across diverse disease categories. SFN exerts its effects through well-characterised pathways, including the Keap1/Nrf2 axis, which regulates phase II detoxification enzymes, and epigenetic mechanisms such as histone deacetylase inhibition. This review evaluates clinical trials registered on ClinicalTrials.gov, focusing on those using SFN or broccoli-derived extracts.

As a result, we identified 84 trials, of which 39 have been published. Results suggest SFN’s potential in regulating redox and inflammatory pathways, improving metabolic and cardiovascular outcomes, and exerting anti-cancer and neuroprotective effects. For healthy subjects, SFN enhanced detoxification and reduced inflammation. In cancer patients, SFN showed promise in early-stage prostate and breast cancer, particularly in GSTM1-positive individuals, but had limited effects in advanced cases. For brain disorders, SFN demonstrated symptomatic improvements in autism spectrum disorder and cognitive benefits in schizophrenia but lacked robust biomarker integration. SFN had minimal impact on respiratory diseases but showed supportive roles in allergic rhinitis therapy. Metabolic disease studies revealed glycaemic control improvements in type 2 diabetes but no benefits for hypertension. Approximately 50% of completed trials remain unpublished, raising concerns about publication bias. While published results highlight SFN’s therapeutic potential, limited sample sizes and inconsistent outcomes underscore the need for more extensive, stratified trials. This review emphasises the importance of integrating mechanistic insights and precision medicine approaches to maximise SFN’s clinical utility.

## Introduction

In the past decade, many epidemiological and clinical research publications have suggested that daily food intake plays a role in the prevention of common diseases such as cancers, cardiovascular conditions, metabolic diseases, and brain disorders^(1–4)^. Such beneficial effects are likely to come from specific nutrients and chemicals included in daily food^(5)^. One of these promising chemicals may be sulforaphane (SFN), which was first isolated from hoary cress and other plants in the mid-20th century. Importantly, glucoraphanin is consumed in daily meals as it is a component of cruciferous vegetables (cauliflower, cabbage, kale, and broccoli). SFN is the product as a result of the hydrolysis of glucoraphanin by myrosinase^(6)^.

SFN is an active phytochemical found within the isothiocyanate group^(7)^ and is a product of its precursor glucoraphanin (*alias* sulforaphane glucosinolate), which is hydrolysed by a thioglucosidase enzyme, myrosinase^(8)^. Although SFN was identified initially many years ago, its biological implication became known in 1992^(6)^ when SFN was isolated from broccoli (*Brassica oleracea italica*). SFN is a significant inducer of phase II detoxification enzymes via the Kelch-like ECH-associated protein-1/nuclear factor erythroid 2-related factor 2 (Keap1/Nrf2) pathway. SFN interacts with Keap1, which releases Nrf2 from the Keap1/Nrf2 complex, allowing Nrf2 to be a functional transcription factor for phase II detoxification enzymes^(9)^. Major genes transcriptionally regulated by Nrf2 include NAD(P)H quinone dehydrogenase 1 (NQO1), heme oxygenase 1 (HO-1), quinone reductase, and glutathione *S*-transferases (GST), as well as inducible nitric oxide synthase^(10)^.

SFN can also interfere with signalling pathways involved in inflammation, such as nuclear factor-kappa B^(11)^. SFN also reportedly inhibits the activity of histone deacetylases (HDACs)^(12)^ and DNA methyltransferases^(13,14)^, respectively, influencing the epigenetic mechanisms and suppression of tumour growth.

As briefly described above, SFN acts through well-defined mechanisms underlying many (or most) cells and organs in the body. Accordingly, clinical trials have taken place to evaluate the effect of SFN on a wide range of disorders, from cancers to brain disorders. Furthermore, since SFN and its precursor, glucoraphanin, can be easily consumed from vegetables, a substantial number of clinical trials using SFN or broccoli sprout on healthy subjects are also available. Nevertheless, to our knowledge, there has been no investigation considering both unpublished and published clinical trials together. To address this knowledge gap, we aimed to examine clinical trials registered in ClinicalTrial.gov (https://clinicaltrials.gov/ct2/home) and compare the clinical trial status of each disease category.

## Selection of clinical trials

**Fig. 1.**
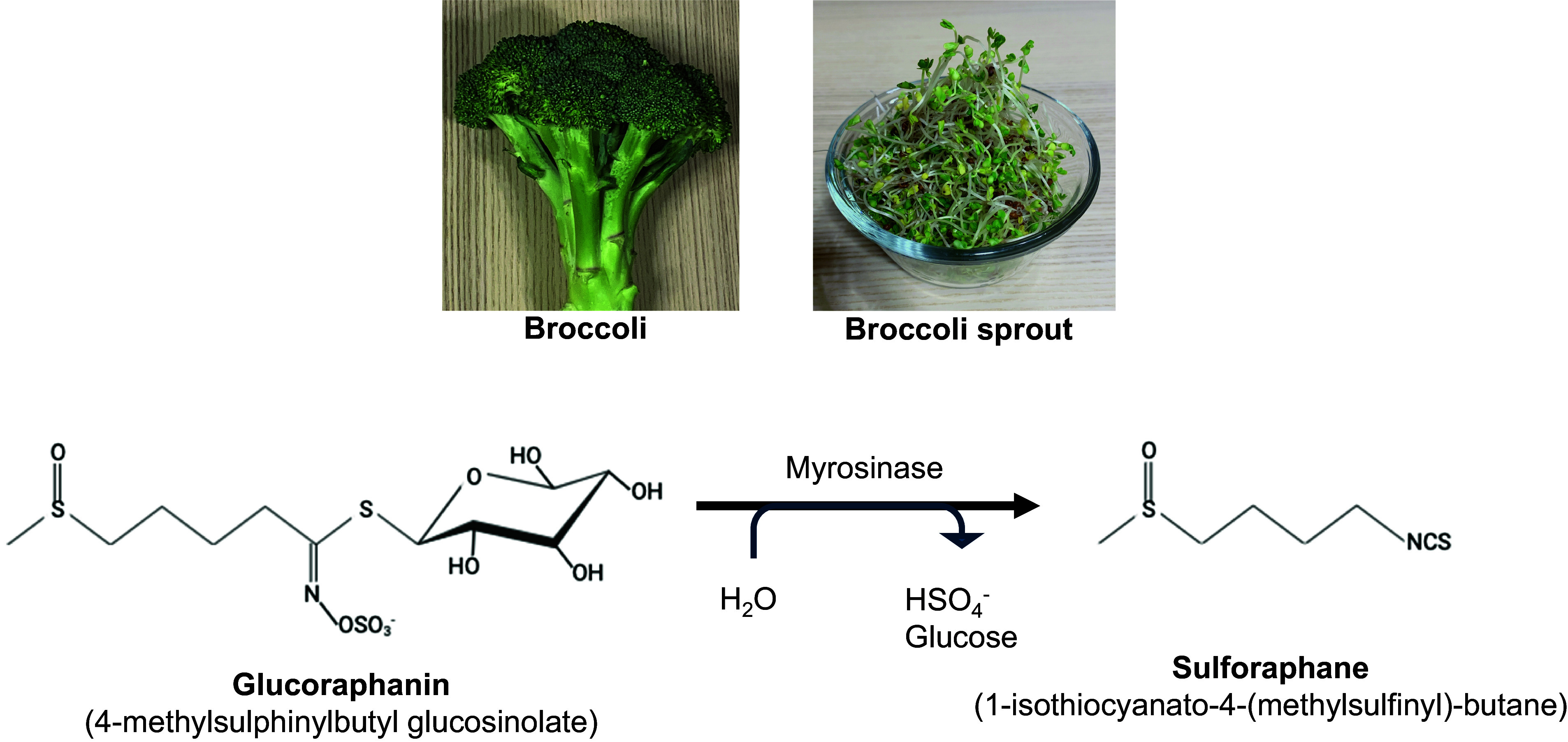
Biosynthesis of sulforaphane (SFN). Glucoraphanin, a type of glucosinolate found in cruciferous vegetables such as broccoli sprouts, is hydrolysed when the plant is damaged. The enzyme myrosinase interacts with glucoraphanin, resulting in the formation of SFN, a beneficial isothiocyanate.

SFN is an organosulfur compound that contains isothiocyanate^(7)^. SFN becomes available when its precursor, glucoraphanin, is hydrolysed by the enzyme myrosinase under neutral pH in cruciferous vegetables; broccoli is known as a common dietary source for SFN (Figure 1).

**Fig. 2.**
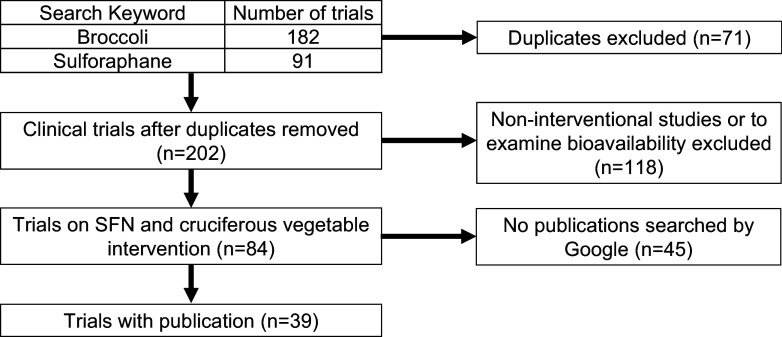
Scheme for clinical trial inclusion. Based on the search result on ClinicalTrials.gov as of June 2024.

The database/literature search process is shown in Figure 2. To narrow the study records, we first filtered the ClinicalTrials.gov database (https://clinicaltrials.gov/  ) by using ‘broccoli’ or ‘sulforaphane’ as a keyword. Consequently, we found 182 and 91 trials for ‘broccoli’ and ‘sulforaphane’ respectively. By comparing these two lists, we found that 71 trials were duplicated, resulting in 202 unique clinical trials. We then carefully examined the content of these 202 trials and chose the target studies based on the following criteria. Inclusion criteria were (1) interventional studies with food or supplement and (2) studies to examine clinical effects, including symptoms and biomarkers. Exclusion criteria were (1) non-interventional study or (2) studies to examine only bioavailability or distribution of the metabolites. As a result, we identified 84 clinical trials that met these criteria. Thus, to explicitly address the effects of SFN, we decided to focus on these 84 trials.

To find which of these 84 trials had been published, we used the clinical trial number (NCT number) from each of these trials as a keyword on Google search (https://www.google.com/webhp). Notably, 39 trials have been successfully published in peer-reviewed journals (Table 1).

**Table 1.** Target conditions of clinical trials

Condition	# of CTs	# of CTs with publication	publication rate (%)
Healthy condition	29	15	51.7
Cancer	20	7	35.0
Brain disorder	19	7	36.8
Respiratory disease	5	4	80.0
Metabolic and cardiovascular disease	3	2	66.7
Infection	2	1	50.0
Miscellaneous disease	6	3	50.0
Total	84	39	46.4

### Trials on healthy conditions

Of the 29 trials on healthy conditions, 15 were published (Table 2). Four trials assessed redox signalling outcomes under the Keap1/Nrf2 pathway, showing that SFN regulated redox markers such as NQO1 and HO-1. For example, broccoli sprout consumption reduced intracellular pro-inflammatory signalling (e.g. P38 MAP kinase) and reactive oxygen species in leukocytes^(15)^. Another trial showed broccoli sprout extract increased NQO1 mRNA in buccal cells, suggesting a chemopreventive role against oral cancer^(16)^. However, a proof-of-concept study revealed that SFN intake failed to mitigate neutrophilic airway inflammation or improve redox markers in peripheral blood mononuclear cells (PBMCs) or nasal epithelial cells after ozone exposure, despite SFN upregulation^(17)^.

**Table 2.** Trials for healthy conditions

NCT#	Outcomes	Target condition	Target subjects	# of subjects assigned (analysed)		Stratification			Main findings by treatment		Mechanisms			Reference
					Dose and duration	Genotype marker	Biopsy, blood, other biofluid marker	Demographic marker	Molecular marker	Clinical progression	R	I	E	
NCT01357070	Significant	Healthy condition	Healthy subjects	6	200g of homogenised broccoli sprouts (BSH) or 200g alfalfa sprouts (ASH, lacking sulforaphane) over 24 hr				Attenuation of intracellular ROS and p38 MAP kinase		✓	✓		15
NCT02023931	Significant	Healthy condition	Healthy subjects	10(9)	600µmol of GR, 150µmol of SFN, or 150µmol topical SFN/daily for 5 days				NQO1 mRNA ↑		✓			16
NCT01625130	Non-Significant	Healthy condition	Healthy subjects	16	200g of BSH/daily for 3 days				No changes in antioxidant gene expression in NEC and PBMC		✓	✓		17
NCT00882115	Significant	Healthy condition	Healthy subjects	29	100µmol of SFN/daily for 4 days	GSTP1 IIe105VaI, GSTM1				Total WBC cell counts in the nasal lavage ↓, no correlation with the genotypes		✓		18
NCT01269723	Significant	Healthy condition	Healthy subjects	51	200g of BSH/daily for 4 days			Smoker or non-smoker	IL-6↓ Influenza B↓ RNA in NLF cells; NQO1 significantly ↑ (of smokers only)		✓	✓		19
	Significant	Healthy condition	Healthy subjects	29	200g of BSH/daily for 4 days			Non-smoker	Granzyme B↑ in NK cells of non-smokers			✓		20
NCT03390855	Significant	Healthy condition	Healthy subjects	40	30g of raw, fresh BS/daily for 70 days			BMI 24.9–29.9	IL-6 ↓ (intervention+ follow-up),CRP ↓ (intervention)	No changes in BW, BMI, Body fat mass↓ (intervention)		✓		21
NCT05146804	Significant	Healthy condition	Healthy subjects	12(11)	16g broccoli sprouts (single intake)				CCL-2 ↑ significantly; sICAM-1, sVCAM-1, hs-CRP, and IL-10 ↑ non-significantly			✓		22
	Significant	Healthy condition	Healthy subjects	12	16g broccoli sprouts (single intake)	GSTM1, GSTP1, GSTT1, NQ01, CYP1A2, UGT1A1, NAT2			Urinary 11-dehydro-TXB2 levels ↓, SNPs in NQO1 gene was correlated with SFN excretion, but not with 11-dehydro-TXB2 levels			✓		23
	Significant	Healthy condition	Healthy subjects	12	16g of sprouts (25 mg of SFN) or placebo followed over 90 min by the standardised high-calorie drink				↓ RMSSD, pNN50, HF↓, hs-CRP ↑, hs-CRP correlates with HRV	Vagal withdrawal and sympathetic dominance		✓		24
NCT01543074	Significant	Healthy condition	Healthy subjects	10; 28	200µmol of SFN/daily for 7 days; low cruciferous vegetables (0–1 serving/week) vs. high (≥5 servings/week)			low cruciferous vegetables (0–1 serving/ week, n = 5) and high (≥5 servings/ week, n = 23)	p16↑, HDAC3↓ in PBMC (200µmol of SFN or high servings)		✓		✓	25
NCT02592954	Significant	Healthy condition	Healthy subjects	5	500nmol/mL of topical SFN/daily for 7 days				KRT17↑, total and phosphorylated NRF2↑		✓			26
NCT01008826	Significant	Healthy condition	Healthy subjects	50	800µmol of GR or 150µmol of SFN/daily for 7 days			Smokers vs. non smokers	Excretion of acrolein conjugate, crotonaldehyde, benzene ↑ in FSR (sulforaphane-rich) and GRR (glucoraphanin-rich) group					27
NCT02656420	Significant	Healthy condition	Healthy subjects	170(169)	600µmol of GR and 40µmol of SFN, 300µmol GR and 20µmol SFN, or 125µmol GR and 8µmol SFN)/daily for 10 days				Excretion of SPMA in urine ↑					28
NCT03402230	Significant	Healthy condition	Healthy subjects	49(48)	148µmol vs. 296 µmol of glucoraphanin/daily for 2 weeks	GSTT1, GSTM1			higher dose significantly ↑ detoxification of benzene, acrolein, and crotonaldehyde; lower dose significantly ↑ detoxification of benzene					29
NCT01437501	Significant	Healthy condition	Healthy subjects	291(267)	600µmol of GR and 40µmol of SFN /daily for 84 consecutive days	GSTT1, GSTM1			Excretion of the glutathione-derived conjugates of benzene, acrolein ↑, (not crotonaldehyde)					30
NCT01114399	Significant	Healthy condition	Healthy subjects	48	400g HG broccoli or 400g standard broccoli/weekly for 12 weeks	PAPOLG (sig), GSTM1 (nonsig)		Sex (males vs. females)	Variation in lipid and amino acid metabolites↓ between PAPOLG genotypes					31
NCT01929564	Significant	Healthy condition	Healthy subjects	130	400g HG (high glucoraphanin) broccoli or 400g standard broccoli/weekly for 12 weeks	GSTM1, PAPOLG, APOE			LDL-C ↓ by standard broccoli, LDL-C ↓↓ by HG broccoli					32

R, redox; I, inflammation; E, epigenetics; and ‘✓’ indicate that the mechanism addressed in the paper. 200g of broccoli sprout homogenate, containing about 100g of fresh broccoli sprout, is estimated to contain approximately 100µmol of SFN^(71,72)^. Mature broccoli is estimated to contain approximately one-tenth the amount of SFN compared to broccoli sprout^(71,72)^. 150µmol of SFN daily is generally not physiologically relevant through diet alone, implying that supplementation is needed to reach these concentrations^(73)^.

Nrf2-independent pathways were also examined. Six trials explored inflammatory outcomes. SFN reduced allergic responses to diesel exhaust, decreasing nasal lavage fluid cells^(18)^. However, it failed to protect against ozone-induced airway neutrophilic inflammation^(17)^. SFN’s anti-inflammatory effects were also evident in virus-exposed individuals, where it enhanced natural killer cell granzyme B production, suggesting improved antiviral defenses^(19,20)^. Interestingly, SFN reduced virus-induced inflammatory markers and viral load in smokers^(19)^. Another trial showed a decrease in body fat mass as well as interleukin 6 and C-reactive protein in the high body mass index group (BMI = 24.9–29.9)^(21)^. Three interrelated publications demonstrated that SFN mitigated caloric load-induced inflammation, improved platelet function, and enhanced heart rate variability in crossover trials^(22–24)^.

Epigenetic modulation was studied in one trial, where cruciferous vegetable intake decreased HDAC3 activity and increased the tumour suppressor gene p16 in PBMCs and colon biopsy samples^(25)^. Another trial demonstrated that topical application of broccoli extract protected the skin and may help manage keratin-based disorders^(26)^. Several trials showed that broccoli sprout consumption increased urinary excretion of toxic carcinogens, supporting detoxification benefits^(27–30)^. Two cardiovascular disease-related trials found that high-glucoraphanin broccoli significantly lowered low-density lipoprotein cholesterol and improved mitochondrial function. Genetic factors, such as the poly(A) polymerase genotype, influenced these effects^(31,32)^.

### Trials on cancers

Seven of 20 cancer-related trials were published (Table 3). Prostate cancer studies revealed SFN altered oncogenic gene expression in prostate tissue but did not reduce plasma prostate-specific antigen levels^(33–36)^. Interestingly, SFN’s effects were more pronounced in glutathione S-transferase mu 1 (GSTM1)-positive patients, suggesting genetic variability impacts therapeutic outcomes. The GSTM1 null genotype, which is prevalent globally, could diminish SFN’s effects^(37)^.

**Table 3.** Trials for cancers

NCT#	Outcomes	Target condition	Target subjects	# of subjects assigned (analysed)		Stratification			Main findings by treatment		Mechanisms			Reference
					Dose and duration	Genotype marker	Biopsy, blood, other biofluid marker	Demographic marker	Molecular marker	Clinical progression	R	I	E	
NCT00535977	Significant	Prostate cancer	Patients	22(20)	400g of high glucosinolate variety broccoli/weekly for 12 months	GSTM1			Moduration of TGFβ1, EGF↑, and insulin signalling in GSTM1 positive group, no changes in PSA					33
NCT01228084	Non-Significant	Prostate cancer	Patients	20(16)	200μmoles/day of sulforaphane-rich extracts/daily up to 20 weeks	GSTM1			no changes in PSA				✓	34
NCT01950143	Significant	Prostate cancer	Patients	61(48)	72 ± 2.8, 214 ± 7.3, or 492 ± 3.2µmol of GR/weekly for 12 months	GSTM1		low-risk or intermediate risk prostate cancer	Oncogenic pathways↓					35
NCT01265953	Significant	Prostate cancer	Patients	98	200µmol of SFN/daily for 4–8 weeks (until prostate biopsy)				ARLNC1↓, AMACR↓in cancer, nomalized by BSE				✓	36
NCT00843167	Significant	Breast cancer	Patients	54	180mg of GR/daily for 8 weeks			benign, ductal carcinoma in situ (DCIS), or invasive ductal carcinoma (IDC)	Ki-67 ↓, HDAC3 ↓ in benign tissue				✓	38
	Significant	Breast cancer	Patients	54	Diet and cruciferous vegetable intake was assessed using Questionnaires			benign, DCIS, or IDC	Ki-67 ↓in DCIS only, non significant HDAC and other biomarkers				✓	39
NCT01753908	Non-Significant	Breast cancer	patients	30(29)	BSE including 200µmol of isothiocyanates/daily for 2 weeks			DCIS, or tumour grade, ER, HER2, PR; breast cancer at any stage, post-menopausal	cleaved caspase3↑, TILs↑, Ki-67↓, ER-α↓ (but not significant-Table 4)				✓	40
NCT01879878	Non-Significant	Pancreatic cancer	Patients	40	508µmol of SFN and 411µmol of GR/daily up to 1 year					Lower death rate at 6mo, higher drop-out rate at 1y compare to placebo				41

R, redox; I, inflammation; E, epigenetics; and ‘✓’ indicate that the mechanism addressed in the paper. 200g of broccoli sprout homogenate, containing about 100g of fresh broccoli sprout, is estimated to contain approximately 100µmol of SFN^(71,72)^. Mature broccoli is estimated to contain approximately one-tenth the amount of SFN compared to broccoli sprout^(71,72)^. 150µmol of SFN daily is generally not physiologically relevant through diet alone, implying that supplementation is needed to reach these concentrations^(73)^.

In breast cancer, two of six registered trials were published. Early-stage patients (ductal carcinoma in situ) showed decreased HDAC activity and reduced cell proliferation, but no benefits were observed in progressive cases^(38–40)^. SFN increased caspase-3 activity and reduced Ki-67 expression, suggesting anti-cancer activity. A trial on advanced pancreatic cancer showed no impact on patients’ overall function^(41)^, potentially due to Nrf2’s dual role in cancer progression depending on genetic mutations^(42)^. These findings underscore the need for subgroup-specific studies considering tumour type, stage, and genetic context.

### Trials on brain disorders

Seven of 19 trials on brain disorders were published (Table 4), including autism spectrum disorder (ASD), fragile-X-associated tremor/ataxia syndrome (FXTAS), and schizophrenia (SZ). ASD trials had relatively high publication rates, with four out of six trials published. The first study (2014) demonstrated clinical improvements with SFN treatment, but subsequent studies reported inconsistent results, including caregiver-rated improvement without significant changes in clinical scores^(43–49)^. One study linked SFN treatment to redox and inflammatory marker changes in PBMCs, though clinical benefits were modest^(46)^. Another trial observed social and behavioural improvements on clinician-rated scales^(48,49)^.

**Table 4.** Trials for brain disorders

NCT#	Outcomes	Target condition	Target subjects	# of subjects assigned (analysed)		Stratification			Main findings by treatment		Mechanisms			Reference
					Dose and duration	Genotype marker	Biopsy, blood, other biofluid marker	Demographic marker	Molecular marker	Clinical progression	R	I	E	
NCT01474993	Significant	Autism spectrum disorder	Patients	44(40)	50, 100, or 150µmol of SFN (adjusted according to the participants’ weight) /daily for 18 weeks			young men (aged 13–27) with moderate to severe ASD		ABC↓, SRS↓, CGI-I↓ (social interaction, abnormal behavior, and verbal communication) (improvements)				43
	Significant	Autism spectrum disorder	Patients	16(9)	9 out of 16 participants still taking SFN supplements					Caregiver rating ↑				44
NCT02654743	Significant	Autism spectrum disorder	Patients	15	222, 259, 296, 333, 370, 444, or 481µmol of GR (adjusted according to the participants’ weight)/daily for 12 weeks.			Children and young adults (ages 5–22, grades K-12)	77 urinary metabolites were identified as significantly correlated with clinical improvements treated with sulforaphane	SRS↓ significantly				45
NCT02561481	Significant	Autism spectrum disorder	Patients	10(6)	2.2µmol of SFN (adjusted according to the participants’ weight) /daily for 2 weeks			10 young males, 6–12.5 years of age	cytoprotective enzymes (NQO1, HO-1, AKR1C1) ↑, heat shock proteins (HSP27, HSP70) ↑ pro-inflammatory markers(IL-6, IL-1β, COX-2, TNF-α)↓	Caregiver rating ↑ (2/10)	✓	✓		46
	Significant	Autism spectrum disorder	Patients	57(45)	45, 60, 90, 105, or 120µmol of SFN (adjusted according to the participants’ weight)/daily for 15 - 30 weeks			Children ages 3–12 years over 36 weeks	significant ↓ IL6,TNF-α, HSP70, HO-1, (free) fGSH/fGSSG, (total) tGSH/tGSSG; significant ↑ mitochondrial function (↑ATP linked respiratory)	significantly ↓ ABC (secondary outcome met), non-significantly ↓ OAIS, SRS-2 (primary outcomes not met)	✓	✓		47
NCT02879110	Significant	Autism spectrum disorder	Patients	17	≥30μmol of glucoraphanin per tablet (adjusted according to the participants’ weight)/daily for 12 weeks.			Boys (4- to 7-years-old)		significantly improve in OSU-CO scores; no change in gut microbiota				48
	Significant	Autism spectrum disorder	Patients	108(53)	24, 36, 48, 72, 84 and 96µmol of GR (adjusted according to the participants’ weight)/daily for 12 weeks.			Children (ages 3–15 years)		Clinician rating significantly ↑ CGI-I and OARS-4 scales (secondary outcome)				49
NCT05233579	Non-Significant	Fragile-X-associated tremor and ataxia syndrome	Patients	11	Avmacol® was increased every other day by 1 tablet to 6 tablets/day for 24 weeks	FMR1	FMR1, FMRP, mitochondrial complex IV in NDEVs	Premutation with FMR1, probable FXTAS or definite FXTAS and FXTAS stages 2–5	Non-significant ↑ in FMRP and mitochondrial complex IV	Non-significant ↑ in MoCA and BDS scores				50
NCT01716858	Significant	Schizophrenia	Patients	10(7)	30mg of SFN-glucosinolate/daily for 8 weeks		BDNF serum levels	aged between 20 and 65 years of age		CogState↑ significantly (Accuracy, Learning), No changes in PANSS				51
NCT02810964	Non-Significant	Schizophrenia	Patients	64(58)	222µmol of GR/daily for 16 weeks			age 18–65		No changes in PANSS (primary outcome), MCCB (secondary outcome)				52

R, redox; I, inflammation; E, epigenetics; and ‘✓’ indicate that the mechanism addressed in the paper. 150 µmol of SFN daily is generally not physiologically relevant through diet alone, implying that supplementation is needed to reach these concentrations^(73)^.

One FXTAS trial did not show improvement in behavioural scores or molecular markers with SFN treatment^(50)^.

Two SZ studies reported no improvements in core symptoms but identified cognitive benefits, particularly in smaller cohorts^(51,52)^. Although redox imbalance and inflammation are implicated in ASD and SZ^(53,54)^, most trials lacked biomarker analyses. Future studies should correlate molecular markers with clinical outcomes.

### Trials on respiratory diseases

Four of five respiratory trials were published (Table 5). SFN had minimal effects on pulmonary function or inflammation in chronic obstructive pulmonary disease^(55)^ or asthma^(56,57)^. For example, two trials reported no significant redox or anti-inflammatory changes after SFN supplementation^(55,57)^. However, in allergic rhinitis, broccoli sprout extract combined with nasal steroids enhanced therapeutic effects, improving peak nasal inspiratory flow and reducing symptom scores^(58)^. These findings suggest SFN may support existing respiratory therapies rather than act as a standalone treatment.

**Table 5.** Trials for respiratory diseases

NCT#	Outcomes	Target condition	Target subjects	# of subjects assigned (analysed)		Stratification			Main findings by treatment		Mechanisms			Reference
					Dose and duration	Genotype marker	Biopsy, blood, other biofluid marker	Demographic marker	Molecular marker	Clinical progression	R	I	E	
NCT01335971	Non-Significant	Chronic obstructive pulmonary disease	Patients	89	25 or 150µmol of SFN/daily for 1 month				No changes in antioxidant (Nrf2 target gene expression) and inflammation in AM and BEC	No changes in pulmonary function tests	✓	✓		55
NCT00994604	Significant	Bronchial Asthma	Patients	45(44)	100μmol of SFN/ daily for 14 days				Increase of NQO1 gene expression by SFN is correlated with increased FEV_1_	Ameliorate Mch effects on FEV1 in 60% of participants, significant decrease in specific airway resistance, increase in small and medium airway luminal area	✓			56
NCT01183923	Non-Significant	Bronchial Asthma	Patients	40	100g of BS/daily for 3 days				No changes in antioxidant gene expression in NEC and PBMC	No changes in FENO and lung function	✓	✓		57
NCT02885025	Significant	Allergic Rhinitis	Patients	47(45)	60–70µmol of SFN/daily for 3 weeks	GSTM1, GSTT1, GSTP1			No significant changes in various cytokines (IL-1, IL-4, IL-5, IL-6, IL-8 and IL-13)	PNIF ↑, TNSS↓		✓		58

R, redox; I, inflammation; E, epigenetics; and ‘✓’ indicate that the mechanism addressed in the paper. 200g of broccoli sprout homogenate, containing about 100g of fresh broccoli sprout, is estimated to contain approximately 100µmol of SFN^(71,72)^. Mature broccoli is estimated to contain approximately one-tenth the amount of SFN compared to broccoli sprout^(71,72)^. 150µmol of SFN daily is generally not physiologically relevant through diet alone, implying that supplementation is needed to reach these concentrations^(73)^.

### Trials on metabolic and cardiovascular diseases

Two of three metabolic and cardiovascular trials were published (Table 6). SFN supplementation did not improve hypertensive patients’ blood pressure or vascular function^(59)^. However, it significantly reduced fasting blood sugar and haemoglobin A1C levels in overweight type 2 diabetes patients, with serum SFN levels correlating with glycaemic improvements^(60)^. Mechanistic insights, such as Nrf2 activation, were demonstrated in rodent studies but remain unexplored in human trials. Future research should investigate SFN’s effects on human metabolism and lipid regulation.

**Table 6.** Trials for metabolic and cardiovascular diseases

NCT#	Outcomes	Target condition	Target subjects	# of subjects assigned (analysed)		Stratification			Main findings by treatment		Mechanisms			Reference
					Dose and duration	Genotype marker	Biopsy, blood, other biofluid marker	Demographic marker	Molecular marker	Clinical progression	R	I	E	
NCT00252018	Non-Significant	Hypertention	Patients	40	10g of dried BS (equivalent to 100g of fresh sprouts)/daily for 4 weeks					No changes in BP (blood pressure), FMD				59
NCT02801448	Significant	Type 2 Diabetes Mellitus	Patients	97	150µmol of SFN/daily for 12 weeks		HbA1c, fasting Glc	Obese vs. non-obese	HbA1c ↓, DHbA1c ↓, Fasting blood glucose↓ in high HbA1c group					60

R, redox; I, inflammation; E, epigenetics. 200g of broccoli sprout homogenate, containing about 100g of fresh broccoli sprout, is estimated to contain approximately 100µmol of SFN^(71,72)^. Mature broccoli is estimated to contain approximately one-tenth the amount of SFN compared to broccoli sprout^(71,72)^. 150µmol of SFN daily is generally not physiologically relevant through diet alone, implying that supplementation is needed to reach these concentrations^(73)^.

### Trials on infectious diseases

One trial evaluated SFN as an adjuvant therapy for *Helicobacter pylori* infection^(61)^ (Table 7). Adding SFN to standard triple therapy did not improve eradication rates or reduce antibiotic-associated adverse events.

**Table 7.** Trials for infectious diseases

NCT#	Outcomes	Target condition	Target subjects	# of subjects assigned (analysed)		Stratification			Main findings by treatment		Mechanisms			Reference
					Dose and duration	Genotype marker	Biopsy, blood, other biofluid marker	Demographic marker	Molecular marker	Clinical progression	R	I	E	
NCT03220542	Non-Significant	H. Pylori infection	Patients	61(53)	1000µg (=5.64µmol) of SFN daily for 4 weeks after clarithromycin-based triple-therapy treatment	CYP2C19				No changes in H. pylori eradication rate and antibiotic-associated adverse events				61

R, redox; I, inflammation; E, epigenetics.

### Trials on miscellaneous diseases

Among six miscellaneous disease trials, three were published (Table 8). Chronic kidney disease studies revealed that SFN upregulated Nrf2 and NQO1 in non-dialysis patients but did not impact oxidative or inflammatory markers in haemodialysis patients^(62,63)^. Another study found no antimicrobial activity against E. coli despite high SFN levels^(64)^. SFN’s effects were also observed in sickle cell disease, where it increased HO-1 and foetal haemoglobin gene expression dose-dependently^(65)^. These findings highlight SFN’s potential benefits in peripheral blood disorders.

**Table 8.** Trials for miscellaneous diseases

NCT#	Outcomes	Target condition	Target subjects	# of subjects assigned (analysed)		Stratification			Main findings by treatment		Mechanisms			Reference
					Dose and duration	Genotype marker	Biopsy, blood, other biofluid marker	Demographic marker	Molecular marker	Clinical progression	R	I	E	
NCT04608903	Significant	Chronic kidney disease	Patients	25	150μmol of SFN/day for 1 month			non-dialysis patients with CKD stages 3–5	Significant ↑ in NRF2, NQO1		✓			62
	Non-Significant	Chronic kidney disease	Patients	25	150μmol of SFN/day for 2 months			regular-dialysis patients for more than 6 months	No significant differences in NRF2, NFKB, TNF-α, and IL-6		✓	✓		63
NCT04113928	Non-Significant	Ileostomy - Stoma	Patients	11	26.5µmol of SFN; with mustard seed: 102µmol of SFN					No inhibitory effects against gut pathogens in ileum				64
NCT01715480	Significant	Sickle cell disease	Patients	15	50, 100, or 150g of fresh BS daily for 21 days	Homozygous for sickel cell			HO-1↑ HBG1↑(trend) in sickle cell		✓			65

R, redox; I, inflammation; E, epigenetics, and ‘✓’ indicate that the mechanism addressed in the paper. 200g of broccoli sprout homogenate, containing about 100g of fresh broccoli sprout, is estimated to contain approximately 100µmol of SFN^(71,72)^. Mature broccoli is estimated to contain approximately one-tenth the amount of SFN compared to broccoli sprout^(71,72)^. 150µmol of SFN daily is generally not physiologically relevant through diet alone, implying that supplementation is needed to reach these concentrations^(73)^.

**Fig. 3.**
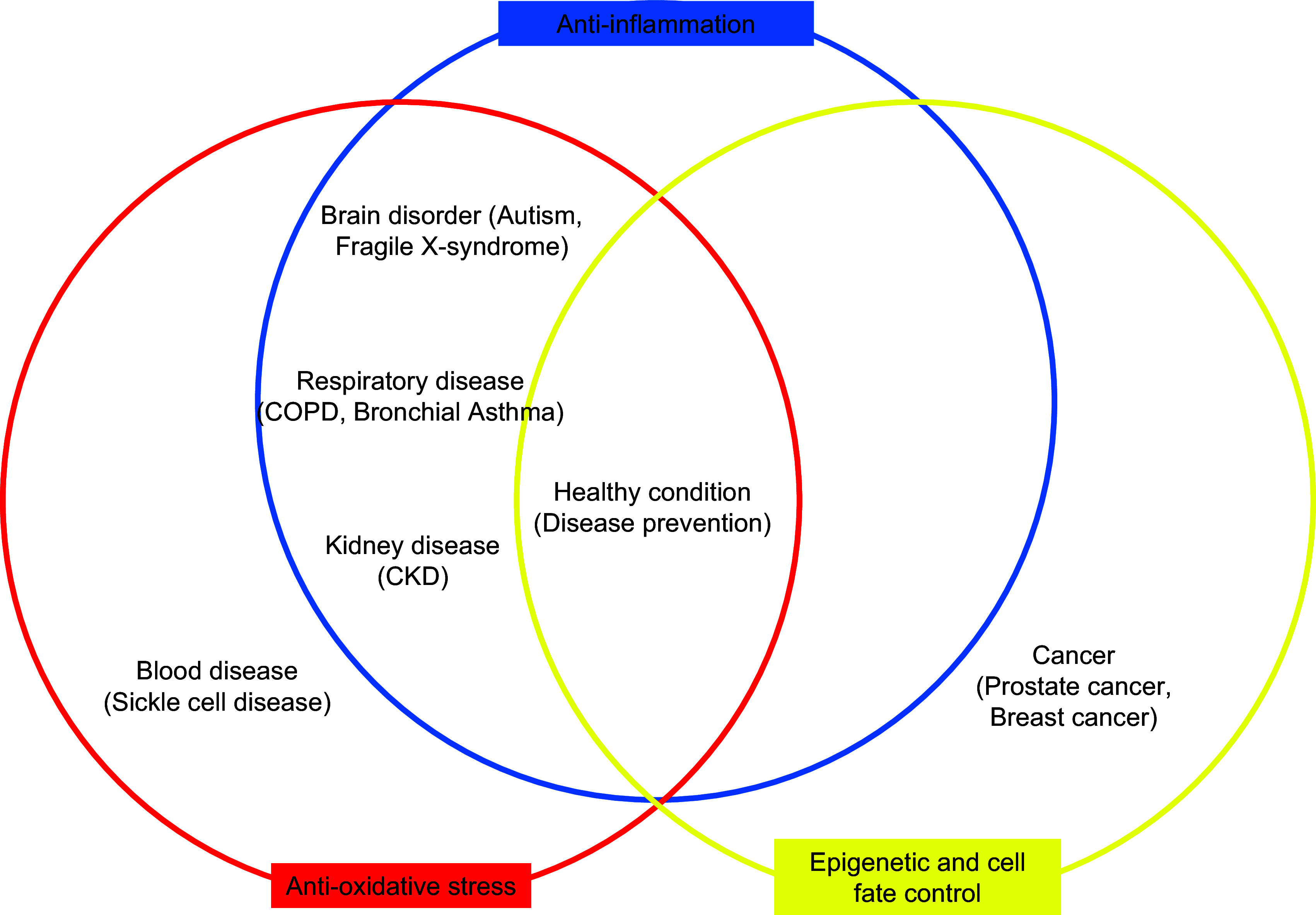
Venn diagram showing sulforaphane mechanisms suggested by the published clinical trials. COPD, chronic obstructive pulmonary disease; CKD, chronic kidney disease.

The major mechanisms underlying SFN’s effects observed in all these studies are summarised in Figure 3.

Additionally, we wish to introduce one study that is not in the database that may help achieve the overall goal of our review. That study examined the effect of SFN on the brain with magnetic resonance spectroscopy^(66)^. It was reported that SFN administration can upregulate glutathione levels in specific brain regions. Ultimately, we may be able to assess the effect of SFN at the mechanistic level in brain disorders in future studies.

## Conclusion and future directions

Numerous clinical trials have investigated the effects of SFN, showing significant benefits across various conditions (100–150 µmol of SFN was mainly used). Although the trials with a single dose (NCT01357070, NCT05146804) showed changes in biomarkers, longer intervention may be required for SFN to have significant clinical effects. However, most of these studies have involved a limited number of participants, and only a few have successfully achieved their primary outcomes. More extensive studies with increased sample sizes are essential to validate these findings. Stratifying participants by specific factors, such as GST genotypes or the severity of clinical stages, has proven effective in identifying populations that are more responsive to SFN. This approach, rooted in the principles of precision medicine, is expected to guide the design of future clinical trials.

We evaluated the number of published studies that show significant changes in outcome measures. Excluding infectious diseases (no publications with substantial changes in outcome measures out of 1 publication [0/1]), the success rate in other groups is 50% or more (Table 9). Given the limited number of publications, making definitive recommendations regarding SFN usage in treating various pathologies is challenging. Notably, about 50% of the completed trials have not been published, and no statistical results are available on ClinicalTrials.gov. This percentage is consistent with the broader issue that only 46% of registered clinical trials are eventually published^(67)^. This low publication rate may suggest that many failed trials remain unreported. Consequently, we focused on unpublished trials with results deposited in the clinical trial database (‘ClinicalTrials.gov’). As no statistical data were deposited for these results, we tested significance using the Mann-Whitney U test. We categorised the trials into two groups: those with and without significant results (*P* < 0.05) (Table 9). The Fisher’s exact test, used to compare the groups (published or unpublished) and the categories (with significance or without significance), did not indicate significant publication bias in the SFN trials (Table 9). However, it is essential to note that data from approximately 40% of completed trials are still unavailable. Continued monitoring of these trials is necessary.

**Table 9.** Publication status and bias

Condition	Published trial	Unpublished trial	Unpublished trial	Mann-Whitney U test P Value
	(Significant outcome/ Non-significant outcome)	(Significant outcome/ Non-significant outcome)	(No results posted)	
Total (*n* = 84)	28/11(*n* = 39)	6/2 (*n* = 8)	*n* = 37	1
Healthy (*n* = 29)	14/1 (*n* = 15)	2/0 (*n* = 2)	*n* = 12	1
Disease (*n* = 55)	14/10 (*n* = 24)	4/2 (*n* = 6)	*n* = 25	1
Cancer (*n* = 20)	4/3 (*n* = 7)	3/0 (*n* = 3)	*n* = 10	0.475
Brain disorder (*n* = 19)	5/2 (*n* = 7)	0/1 (*n* = 1)	*n* = 11	0.375
Respiratory (*n* = 5)	2/2 (*n* = 4)	0/0 (*n* = 0)	*n* = 1	1
Metabolic (*n* = 3)	1/1 (*n* = 2)	0/0 (*n* = 0)	*n* = 1	1
Infectious (*n* = 2)	0/1 (*n* = 1)	0/0 (*n* = 0)	*n* = 1	1
Miscellaneous (*n* = 6)	2/1 (*n* = 3)	1/1 (*n* = 2)	*n* = 1	1

A limitation of this review is that the number of studies listed in this review is relatively smaller than other comprehensive reviews about SFN^(68,69)^. Although we have examined the most authentic and widely used database of clinical trials (‘ClinicalTrials.gov’), some studies may not be included in the database. We acknowledge that there are other databases, such as the International Standard Randomised Controlled Trial Number (ISRCTN) registry, EU Clinical Trials Register, and Pan African Clinical Trial Registry (PACTR). However, they are much smaller in size compared with the ClinicalTrials.gov database. Although another database, the International Clinical Trials Registry Platform (ICTRP), organised by the WHO, is relatively larger, as claimed by the WHO itself, this platform is not endorsed by the WHO. The WHO also stated that the agency is not responsible for the accuracy, completeness, and/or use of the content displayed for any trial record. Furthermore, two-thirds of the studies in this WHO platform are also available in the ClinicalTrials.gov database, addressing the specific topic covered in this review. Altogether, we have decided not to include the information from the ICTRP in our study. Nonetheless, we wish to note that several studies hoping to address the disease-related mechanism of SFN have not been covered in the present search. For instance, the first type 2 diabetes trial from an Iranian group is not included in the ClinicalTrials.gov database^(70)^.

We have reviewed over 80 clinical trials for this study; however, due to the comparison of each disease category, the number of studies in each category is relatively small. Therefore, our statement remains a qualitative comment, which is far from a quantitative statistical analysis. On the other hand, by taking advantage of the fact that the present study encompasses a wide range of disease conditions, spanning from cancers to neuropsychiatric disorders, we propose that SFN may be a useful tool for examining the body-brain connection and that clinical trials with SFN may provide more insight into its biology. This possibility is particularly timely, as the significance of the body-brain connection has been recently highlighted, such as through the concept of the gut-brain axis.

## Data Availability

All relevant data are available upon request to the corresponding authors.
